# pH in human tumour xenografts: effect of intravenous administration of glucose.

**DOI:** 10.1038/bjc.1993.375

**Published:** 1993-09

**Authors:** T. Volk, E. Jähde, H. P. Fortmeyer, K. H. Glüsenkamp, M. F. Rajewsky

**Affiliations:** Institute of Cell Biology (Cancer Research), West German Cancer Center Essen, University of Essen Medical School.

## Abstract

pH frequency distributions of tumours grown s.c. from 30 human tumour xenograft lines in rnu/rnu rats were analysed with the use of H+ ion-sensitive semi-microelectrodes prior to and following stimulation of tumour cell glycolysis by i.v. infusion of glucose. At normoglycemia, the average pH of the tumours investigated was 6.83 (range, 6.72-7.01; n = 268). Without exception, all xenografts responded to the temporary increase in plasma glucose concentration (PGC) from 6 +/- 1 to 30 +/- 3 mM by an accumulation of acidic metabolites, as indicated by a pH reduction to an average value of 6.43 (range, 6.12-6.78; n = 292). This pH value corresponds to a ten-fold increase in H+ ion activity in tumour tissue as compared to arterial blood. Tumour pH approached minimum values at 2-4 h after the onset of glucose administration and could be maintained at acidic levels for 24 h by controlled glucose infusion. Irrespective of pH variations between tumours grown from individual xenograft lines, there was no major difference in pH response to glucose between the four main histopathological tumour entities investigated, i.e. breast, lung and gastrointestinal carcinomas, and sarcomas. In tumours from several xenograft lines, an increase in blood glucose to only 2.5-times the normal value (14 mM) was sufficient to reduce the mean pH to 6.4. Glucose-induced acidosis was tumour-specific. The pH frequency distributions in liver, kidney and skeletal muscle of tumour-bearing rnu/rnu rats were only marginally sensitive to hyperglycemia (average pH, 6.97 vs normal value of 7.14). Tumour-selective activation of pH-sensitive anti-cancer agents, e.g. alkylating drugs, acid-labile prodrugs or pH-sensitive immunoconjugates may thus be feasible in a wide variety of human cancers.


Br. J. Cancer (1993), 68, 492–500 © Macmillan Press Ltd., 1993

### pH in human tumour xenografts: effect of intravenous administration of glucose

T. Volk[1], E. Jähde[1], H.P. Fortmeyer[2], K.-H. Glüsenkamp[1] & M.F. Rajewsky[1]

[1]*Institute of Cell Biology (Cancer Research), West German Cancer Center Essen, University of Essen Medical School, Hufelandstrasse 55, D-45122 Essen;* [2]*Department of Animal Experimentation, University of Frankfurt a.M., Theodor-Stern-Kai 7, D-60596 Frankfurt am Main, Germany.*

**Summary** pH frequency distributions of tumours grown s.c. from 30 human tumour xenograft lines in rnu/rnu rats were analysed with the use of $H^+$ ion-sensitive semi-microelectrodes prior to and following stimulation of tumour cell glycolysis by i.v. infusion of glucose. At normoglycemia, the average pH of the tumours investigated was 6.83 (range, 6.72–7.01; $n = 268$). Without exception, all xenografts responded to the temporary increase in plasma glucose concentration (PGC) from $6 \pm 1$ to $30 \pm 3$ mM by an accumulation of acidic metabolites, as indicated by a pH reduction to an average value of 6.43 (range, 6.12–6.78; $n = 292$). This pH value corresponds to a ten-fold increase in $H^+$ ion activity in tumour tissue as compared to arterial blood. Tumour pH approached minimum values at 2–4 h after the onset of glucose administration and could be maintained at acidic levels for 24 h by controlled glucose infusion. Irrespective of pH variations between tumours grown from individual xenograft lines, there was no major difference in pH response to glucose between the four main histopathological tumour entities investigated, i.e. breast, lung and gastrointestinal carcinomas, and sarcomas. In tumours from several xenograft lines, an increase in blood glucose to only 2.5-times the normal value (14 mM) was sufficient to reduce the mean pH to 6.4. Glucose-induced acidosis was tumour-specific. The pH frequency distributions in liver, kidney and skeletal muscle of tumour-bearing rnu/rnu rats were only marginally sensitive to hyperglycemia (average pH, 6.97 *vs* normal value of 7.14). Tumour-selective activation of pH-sensitive anti-cancer agents, e.g. alkylating drugs, acid-labile prodrugs or pH-sensitive immunoconjugates may thus be feasible in a wide variety of human cancers.

Any attempt to improve the selectivity of anti-cancer agents must be based on genetic, phenotypic or pathophysiological differences distinguishing cancer cells and tissues from their normal counterparts. Molecular alterations underlying the process of malignant transformation and tumour progression not only comprise mutations, but also changes in transcription of normal genes controlling cellular functions whose dysregulation is critically associated with the expression of malignant phenotypes (Schutzbank *et al.*, 1982; Birnbaum *et al.*, 1987; Busch, 1990). To improve the therapeutic index of anti-cancer agents strategies are thus being sought that would exploit such transformation-associated changes by rational drug design. For example, in malignant cells the expression of genes coding for membrane-based glucose transporters and glycolytic enzymes is frequently increased; this molecular alteration is pivotal to a metabolic hallmark of malignant cells, aerobic glycolysis (Birnbaum *et al.*, 1987; Flier *et al.*, 1987). The capacity of cancer cells to metabolise glucose to lactic acid (Warburg *et al.*, 1924; Aisenberg, 1961; Weber, 1977; Lyon *et al.*, 1988) can be exploited to stimulate lactate production selectively in malignant tissues. This manipulation involves a temporary induction of hyperglycemia in tumour-bearing hosts by i.v. infusion of glucose and results in an increased activity of $H^+$ ions in malignant tissues (Jähde *et al.*, 1982*b*; Wike-Hooley *et al.*, 1984; Ward & Jain, 1988; Jähde *et al.*, 1989; Hwang *et al.*, 1991). Since $H^+$ ions are potent modulators of various types of chemical reactions, tumour-selective acidification can be made use of, in a second step, to increase the target cell-directed cytotoxicity of anti-cancer agents, e.g. alkylating drugs, acid-labile prodrugs, pH-sensitive immunoconjugates, hyperthermia, or acid-labile liposomes (Jähde *et al.*, 1989; Tietze *et al.*, 1989; Lavie *et al.*, 1991; Hiroaka & Hahn, 1989; Yatvin *et al.*, 1980).

The pathophysiological basis of this approach has so far been investigated almost exclusively in transplanted rodent tumours. With the use of invasive as well as noninvasive techniques for measuring pH, the $H^+$ ion activity in a wide variety of animal tumours was shown to be increased following stimulation of aerobic glycolysis (Wike-Hooley *et al.*, 1984; Ward & Jain, 1988; Jähde *et al.*, 1982*b*; Jähde *et al.*, 1989). We are, however, only aware of few investigations analysing the $H^+$ ion activity in human tumours *in situ* following glucose-mediated stimulation of lactic acid production (Naeslund & Swenson, 1953; Ashby, 1966; Thistlethwaite, 1987; Lavie *et al.*, 1991). In these studies, only 25 individual tumours, including ten malignant melanomas, five female genital cancers and ten tumours of varying histology were analysed. To establish the prerequisites for clinical trials with pH-sensitive cytotoxic agents, we are investigating whether the response of human tumours to stimulation of aerobic glycolysis is similar to that of transplanted rodent tumours. As a first step toward this goal we have measured the $H^+$ ion activity in a large panel of human tumour xenografts with and without induction of hyperglycemia. In particular, we have investigated (i) whether there are histopathological tumour entitites of major clinical importance which are not responsive to glucose, and (ii) to what extent individual tumours vary in their pH response. For these studies, 30 human tumour xenograft lines of diverse histogenesis were established in congenitally athymic rats. A further objective of this work has been to define the level of hyperglycemia required to reduce the pH in human tumour tissues to values compatible with tumour-selective activation of cytotoxic agents (i.e. ~0.5 pH units below the value of normal tissues). In addition, the accumulation of acidic metabolites in human tumour xenografts was investigated as a function of tumour mass, tumour growth rate, and the duration of hyperglycemia.

### Materials and methods

### *Animals*

Congenitally athymic rats (body weight, $220 \pm 20$ g) were used since pilot experiments had indicated that nude mice were, for technical reasons, less suited for long-term i.v. infusion via implanted central venous catheters. Male and female Han:RNU-rnu/rnu rats, 4–6 weeks old, were purchased from Zentralinstitut für Versuchstierzucht (Hannover, Germany) and LEW/Mol-rnu/rnu rats of the same age from Møllegaard Breeding Center (Ejby, Denmark). Rats were

Correspondence: M.F. Rajewsky.
Received 2 February 1993; and in revised form 4 May 1993.

housed in groups of three on sterile bedding in isolator cabinets (Altromin, Lage, Germany) at 24°C, 60–70% humidity and a 12-h light-dark cycle. Animals were fed type 1434 diet (Altromin, Lage, Germany) and had free access to sterile acidified water (pH 3) supplemented with chlorotetracycline (3 g $l^{-1}$).

*Tumours*

Human tumour xenograft lines, BL, BÖ, BR, CH, GÄ, GE, JE, KO, LA, REI, SE (breast), SE (lung), SP, SCHRÖ, and STO, previously grown in nu/nu mice, were re-established from frozen tissue samples in this species for one passage before transplantation into rnu/rnu rats. Nude mice bearing the s.c. implanted xenograft lines CXF 1103, LXFA 289, and LXFE 211 were kindly provided by Dr H.H. Fiebig (Department of Internal Medicine I, University of Freiburg i.Br., Germany); mice transplanted with SCLC and SW 707 xenografts by Dr K. Wayss (German Cancer Research Center, Heidelberg, Germany); and mice bearing N4 and F8 tumours by Dr V. Budach (Department of Radiotherapy, University of Essen Medical School, Essen, Germany). Tumour lines H-MESO, MRI-H-221, MRI-H-121B, MX-1, and LX-1, were obtained as frozen tissue samples from the Tumour Bank of the German Cancer Research Center, Heidelberg, and transplanted directly into rnu/rnu rats. Xenografts A549, WiDr, and 791/M were established by s.c. injection of $5 \times 10^6$ cultured cells (A549 and WiDr, respectively, American Type Culture Collection, Rockville, MD, USA; 791/M, Xoma, Berkeley, CA, USA) into rnu/rnu rats. The histopathological classification of the xenograft lines is presented in Figures 1 and 3. When mouse xenografts had reached a size of $\sim 1\ cm^3$, animals were sacrificed and tumour fragments of $\sim 8\ mm^3$ were transplanted s.c. into both flanks of rnu/rnu rats. Routinely, all pH measurements were performed on tumours of the first and second rat passage. Only BR, F8, H-MESO, MRI-H-221, N4, REI, and SCLC xenografts were transplanted serially for up to 14 passages in rnu/rnu rats. At the first rat passage, samples of tumour tissue were fixed in 10% buffered formalin and stained with hematoxylin-eosin. Tumour sections were evaluated microscopically to confirm the histopathological classification communicated by the donors, and to ascertain that the tumour lines studied did not exhibit signs of tissue rejection. Tumour growth was monitored by caliper measurements and experiments were performed 9–61 days (mean, 33 days) following tumour transplantation. Unless otherwise stated, at this time tumour volume ranged between 1.2 and 2.8 $cm^3$.

*Glucose administration*

All infusions were performed i.v. in unanaesthetised, unrestrained rats. Central venous catheters were made of polyethylene tubing (Jähde *et al.*,1982*b*). Under light ether anaesthesia, catheters were advanced into the right atrium via phlebotomy of the right jugular vein and connected to a flexible, freely rotating infusion line fixed to the neck of the animals. The infusion apparatus allowed for free movement of animals in specially designed glass cages (Jähde *et al.*, 1982*b*). Glucose or NaCl solutions were continuously infused with the aid of standard syringe pumps (Braun Melsungen, Melsungen, Germany). Routinely, rats were connected to the infusion apparatus 1 day prior to the experiments. To maintain the patency of the catheters, NaCl solution (9 g $l^{-1}$) was infused at a rate of 0.5 ml $h^{-1}$ until glucose infusion was initiated. The rate at which sterile glucose solution (0.4 g $ml^{-1}$) was infused was adjusted according to the desired PGC. In order to raise PGC to 30 ± 3 mM, the infusion rate was set to 5.0 ml $h^{-1}$ initially, and thereafter adjusted individually for each rat according to PGC determinations at intervals of 0.5–3 h. During steady state hyperglycemia (i.e. $\sim$1 h after onset of glucose infusion; PGC 30 ± 3 mM) the rate of glucose infusion ranged between 2 and 3.5 ml $h^{-1}$ of the above solution, values corresponding to a glucose load of 61–106 mg $kg^{-1}$ body weight $min^{-1}$. Blood glucose concentrations were measured in 20 $\mu$l samples of tail vein blood with the use of an automated glucose analyser (model ESAT 6660, Eppendorf-Netheler-Hinz, Hamburg, Germany). Since the kinetics of pH reduction in human tumour xenografts had not been investigated previously, pH measurements were performed, unless otherwise stated, at 4 h after PGC had reached plateau values.

*pH measurements*

pH values in human tumour xenografts and normal tissues (liver, kidney, skeletal muscle) of rnu/rnu rats were measured with the use of glass electrodes incorporated into a 25 gauge bevelled steel needle (length of the sensing portion, 250 $\mu$m; model 802, Diamond General, Ann Arbor, MI, USA). Due to the large number of measurements, these semi-micro-electrodes, which exhibit high mechanical stability, were preferred over fragile glass microelectrodes (Jähde *et al.*, 1982*a*). As previously discussed, pH measurements performed with electrodes of this size primarily reflect the $H^+$ ion activity in extracellular fluid, with a small but not precisely known contribution by intracellular components (Jähde *et al.*, 1982*a*). Ag/AgCl electrodes (type 373, Ingold, Frankfurt a.M., Germany) were used as reference electrodes. These electrodes were connected to tissues (buffers) via a 2 M KCl interface and an agar bridge prepared by filling a glass capillary with an aqueous solution of NaCl (9 g $l^{-1}$) and agar (10 g $l^{-1}$; heated to 100°C). A Keithley 616 electrometer (Keithley Instruments, Cleveland, OH, USA) was used for signal amplification. The reference unit was tested by connecting a standard pH electrode (type 275; Ingold) to the electrometer. All measurements were performed in an electrically shielded cage. Electrodes were calibrated at 37°C in physiological phosphate buffers (pH 5.00, 6.03, 6.83, 7.15, and 7.33) and recalibrated at intervals of 2 h. Typically, electrodes exhibited the following sensing properties: slope, 58–60 mV $pH^{-1}$ unit; response time (90%), 10–20 s; drift, 0.21 mV $h^{-1}$.

Tumour-bearing rats were anaesthetised with sodium pentobarbital (Nembutal®, 30 $\mu$g $g^{-1}$ body weight; Abbott, Bad Segeberg, Germany) and immobilised on a heating pad. Rectal temperatures were recorded by thermocouples. When required, additional heating was provided by infrared irradiation. Immediately prior to measurements, skin and fibrous tissue overlying tumour surfaces were carefully removed over an area of $\sim 10\ mm^2$. Particular care was taken not to damage blood vessels. The reference capillary was placed on the tumour surface avoiding tissue compression. pH semi-microelectrodes were then automatically inserted to a depth of 5–10 mm (according to tumour size) at a speed of 0.5 mm $min^{-1}$ using mechanically driven remote-control micromanipulators (Jähde *et al.*, 1982*a*; Jähde *et al.*, 1982*b*). To improve representative sampling in tumours of $>2$ g, two electrodes were mounted in parallel (tip distance, 3–4 mm) and used simultaneously.

Electrode signals were continuously recorded on a type SE 130 2-channel pen recorder (ABB Goerz Metrawatt, Wien, Austria). At distances of 0.5 mm, single-point pH values were calculated assuming linear electrode drift between consecutive calibrations. For data analyses, signals were digitalised using a 12 bit A/D converter (Kolter Electronic, Erftstadt, Germany) and transferred to a personal computer. Data acquisition, statistical analysis and graphic display were accomplished with the aid of a specially designed computer programme. Unless otherwise stated, all pH frequency distributions, mean values and ranges refer to measurements in groups of at least eight tumours or to the same number of recordings in normal tissues.

Liver, kidney and skeletal muscle were used as control tissues. The technique used for pH measurements in kidney has been described (Jähde *et al.*, 1982*b*). In preparation of liver, care was taken to tightly fix, by tissue glue (Histioacryl®, Braun Melsungen, Melsungen, Germany), one liver lobe to a specially designed metal tray in order to avoid artifacts caused by respiratory movements. Analyses of skeletal muscle were performed in muscles of the left thigh.

### *Arterial blood parameters*

To monitor side-effects of high-dose glucose infusion on hemodynamics, arterial blood pressure was measured by a pressure-transducer (Peter von Berg, Kirchseeon/Eglharting, Germany) coupled to a monitor (Sirecust 961, Siemens, Erlangen, Germany) via a 24 G cannula inserted into the abdominal aorta. Blood drawn from this cannula after 5 min of pressure registration was analysed for $pO_2$, $pCO_2$, pH, $K^+$, $Na^+$ and hemoglobin with the use of a standard blood gas analyser (model 288 Blood Gas System, Ciba Corning Diagnostics, Fernwald, Germany). In addition, plasma protein concentration was determined by an Ektachem DT60 analyser (Kodak, Stuttgart, Germany).

### *Statistical evaluation*

Descriptive statistical parameters (means, s.d.) were calculated and two-sample *t*-tests were applied to test for differences in two groups. The Bonferroni adjustment was used to adjust for multiple testing. *P* values less than 0.05 were judged to be of significance.

### Results

Thirty human tumour xenograft lines were selected for this study. The composition of the tumour panel reflected, to some extent, the incidence of various human histopathological tumour entities. The panel included seven mammary carcinomas, eight lung cancers, four gastrointestinal tumours, four sarcomas, and seven tumours of diverse histogenesis.

### *$H^+$ ion activity in human tumour xenografts at normal PGC*

At normal PGC (6 ± 1 mM), the average pH of all xenografts investigated was 6.84 ($n = 268$), with extreme single-point readings of 6.44 and 7.45, respectively (Figure 1). In single tumours, local pH values varied within 0.3 to 0.8 units. This broad overall distribution of pH readings resulted from (i) local heterogeneities of $H^+$ ion activity in individual tumours, (ii) inter-tumour pH variations in xenograft lines, and (iii) differences in the pH frequency distributions between various tumour lines (Figure 1). As exemplified by the pH histograms shown in Figures 2–4, the range of pH readings in individual xenograft lines was typically 0.3–0.5 units; the mean pH values of all lines investigated ranged between 6.71 (lung cancer SCHRÖ) and 7.01 (gastric cancer SP) (Figure 1). There was no major difference between the overall pH fre-

**Figure 1** pH in human tumour xenografts at normal PGC. The vertical line at pH 6.84 indicates the average value of all single-point measurements recorded in a total of 268 xenografts. Black bars indicate the difference between the former value and the mean pH of individual tumour xenografts. Of each tumour line, 5–12 individual xenografts were analysed. Hatched area: physiological range of pH values in arterial blood of tumour-bearing rnu/rnu rats.

**Figure 2** Effect of i.v. glucose infusion on $H^+$ ion activity in SW 707 human colon cancer, LA human endometrial carcinoma and CXF 1103 human colon cancer xenografts: frequency distributions of single-point pH readings at **a**, normoglycemia (PGC, 6 ± 1 mM) and **b**, at 5 h after the initiation of glucose infusion (PGC, 30 ± 3 mM). SW 707 colon cancer, **a**, five tumours, number of pH readings, 165; **b**, six tumours, number of pH readings, 204; LA endometrial carcinoma, **a**, six tumours, number of pH readings, 116; **b**, nine tumours, number of pH readings, 276; CXF 1103 colon cancer, **a**, six tumours, number of pH readings, 188; **b**, nine tumours, number of pH readings, 384.

quency distributions of the four main histopathological tumour entities investigated (breast, lung and gastrointestinal carcinomas, and sarcomas). In this sequence, the average pH values of these entities were 6.79, 6.84, 6.93 and 6.87, respectively).

*Effect of i.v. glucose administration on pH in human tumour xenografts*

All tumours grown from the human xenograft lines investigated responded to glucose-stimulated aerobic glycolysis by an increase in intratumoural $H^+$ ion activity. After 5 h of i.v. infusion of glucose (PGC, 30 ± 3 mM), the average pH of all tumours analysed was reduced to 6.43 (range of single-point readings, 5.47–7.16; $n = 292$, $P < 0.0001$) (Figure 3). The pH response of single xenograft lines to glucose varied markedly, with mean values ranging between 6.13 (breast carcinoma SE) and 6.78 (colon cancer CXF 1103) (Figure 3). pH histograms of three representative tumour lines recorded prior to and following induction of hyperglycemia are shown in Figure 2. In general, the glucose-mediated shift of pH histograms to more acidic values was accompanied by a broadening of the pH frequency distributions. This effect was predominantly due to an increase in the range of pH values in all individual tumours of a given xenograft line and not to tumours lacking a pH response to glucose, indicating local heterogeneities in glucose metabolism as well as substrate and metabolite transport. In colon cancer SW707, >90% of all pH readings ranged between 6.8 and 7.0 at normal PGC. During hyperglycemia, however, single-point pH values as low as 5.7 were recorded (mean, 6.45, $P < 0.004$) (Figure 2). Similarly, in normoglycemic endometrial carcinoma LA, the pH frequency distribution encompassed values between 6.4 and 7.0 (mean, 6.79). However, following stimulation of aerobic glycolysis, ~25% of all pH readings were <6.0 (mean, 6.23, $P < 0.004$) (Figure 2). An example of a xenograft line responding to glucose only moderately is given in Figure 2. At elevated plasma glucose, the pH frequency distribution of CXF 1103 tumours was only slightly shifted to more acidic values (mean, 6.78, $P < 0.005$) as compared to the pH histogram recorded at normal PGC (mean value, 6.97).

Irrespective of marked differences between individual xenograft lines, there were only small variations in the overall pH response to glucose among the main tumour entities investigated (Table I). On average, the $H^+$ ion activity in all other tumours investigated was increased by a factor of 3.3 (mean pH, 6.31; normoglycemia: mean pH, 6.83; $P < 0.0001$) and by a factor of 12 as compared to arterial blood.

Pretreatment pH was not predictive of the response to glucose. For example, the activity of acidic metabolites in LXFA 289, a xenograft line exhibiting a low pH at normoglycemia (mean, 6.75), decreased only slightly (mean pH, 6.55; not significant) when PGC was raised to 30 ± 3 mM. Conversely, stomach carcinoma SP, with a mean pretreatment pH near neutrality, responded to glucose by a pH reduction by >0.6 units ($P < 0.0001$, Figures 1,3). In Figure 4, the glucose-mediated pH shift in individual xenograft lines is depicted as a function of $H^+$ ion activity at normal PGC. There was no statistically significant correlation between these parameters (R = 0.205).

*Effect of different levels of hyperglycemia on $H^+$ ion activity in human tumour xenografts*

To investigate the correlation between PGC and tumour pH in more detail, PGC of rnu/rnu rats bearing MRI-H-221 xenografts was elevated to plateau values of 14 ± 3, 19 ± 3, or 30 ± 3 mM, respectively, and maintained at these levels for 4 h prior to pH measurements. The highest PGC was chosen according to studies on human volunteers and cancer patients in whom PGC levels of 30–35 mM have been generated safely by i.v. glucose infusion (Lippmann & Graichen, 1977; Förster, 1987; Krag *et al.*, 1990). An increase in PGC to only about 2.5 times the normal value (14 ± 3 mM) was sufficient to cause an acidic pH shift in MRI-H-221 xenografts from 6.72 to 6.46 ($P < 0.004$; Figure 7). At a PGC of 19 ± 3 mM, the mean intratumoural pH was 6.31 ($P < 0.05$) and a further increase in blood glucose to 27–33 mM only resulted in a small further increment in tumour acidity (mean pH, 6.19; statistically not significant) (Figure 5).

*Dependence of intratumoural pH on duration of hyperglycemia*

To investigate the effect of various durations of hyperglycemia on the accumulation of acidic metabolites in human

**Table I** Effect of hyperglycemia on average pH in human tumour xenografts of four histopathological types

| *Histopathological tumour type* | *pH* Normoglycemia (PGC, 6 ± 1 mM) | Hyperglycemia[a] (PGC, 30 ± 3 mM) |
|---|---|---|
| Breast cancers | 6.79 (6.72–6.88)[b] | 6.33 (6.13–6.56) |
| Lung cancers | 6.84 (6.74–7.38) | 6.53 (6.29–6.71) |
| Gastronintestinal cancers | 6.93 (6.89–7.54) | 6.53 (6.37–6.87) |
| Sarcomas | 6.85 (6.76–6.95) | 6.48 (6.41–6.56) |

[a]In hyperglycemic tumours, measurements were performed at 4 h after PGC had reached a plateau. [b]Mean values with ranges in brackets.

**Figure 3** $H^+$ ion activity in human tumour xenografts following glucose-mediated stimulation of aerobic glycolysis. pH measurements were performed at 4 h after PGC was raised to 30 ± 3 mM. Designations as in Figure 1. Total number of tumours, 292.

**Figure 4** Correlation between pretreatment pH values in human tumour xenografts and pH shifts (Δ pH) induced by glucose-mediated stimulation of aerobic glycolysis (PGC, 30 ± 3 mM). Points represent mean values of single xenograft lines.

**Figure 5** Dependence of $H^+$ ion activity in MRI-H-221 human lung cancer xenografts on the level of hyperglycemia: frequency distributions of single-point pH readings. PGC, 6 ± 1 mM: ten tumours; number of pH readings, 339. PGC, 14 ± 3 mM: nine tumours; number of pH readings, 372. PGC, 19 ± 3 mM: six tumours; number of pH readings, 260. PGC, 30 ± 3 mM: 12 tumours; number of pH readings, 500.

tumour xenografts, a PGC of 30 ± 3 mM was maintained for 2, 4, 7, or 24 h, respectively, in animals bearing SCLC lung carcinomas or N4 sarcomas. In SCLC xenografts, the mean pretreatment pH of 6.89 rapidly fell to 6.36 ($P<0.0001$) at 2 h after PGC had reached a plateau and remained at this level for another 2 h (mean pH at 4 h, 6.39). During the time interval up to 24 h, tumour pH increased slightly to a mean value of 6.46. N4 sarcomas exhibited a somewhat different response to long-term glucose infusion: following initial pH shifts from 6.90 to 6.56 ($P<0.0001$) and 6.51 at 2 and 7 h, respectively, intratumoural acidity thereafter continued to increase slightly. At 24 h the pH frequency distribution exhibited a mean value of 6.45.

*Effect of tumour mass and growth rate on pH in human tumour xenografts*

The growth of malignant tumours is frequently accompanied by changes in various histomorphological parameters governing the vascular and interstitial transport of substrates and metabolites (Vaupel *et al.*, 1989). To investigate the effects of these changes on the accumulation of acidic metabolites, pH values in breast carcinomas REI and mesotheliomas H-MESO were measured as a function of tumour mass (0.6–15.0 g). At normal PGC, the mean pH values in individual tumours of both xenograft lines ranged between 6.5 and 7.0. This distribution of values was not correlated with tumour mass (Figure 6). At hyperglycemia (PGC, 30 ± 3 mM) smaller mesotheliomas tended to be slightly more acidic than larger tumours of the same type (R = 0.45, $P<0.02$) whereas breast cancers REI exhibited an inverse correlation between tumour mass and mean tumour pH (R = − 0.069; statistically not significant; Figure 6). Conversely, the glucose-induced downshift of pH was independent of the growth rate of individual xenograft lines as estimated by the time required to reach a mean tumour volume of 2 $cm^3$ (R = − 0.33).

**Figure 6** Weight-dependence of $H^+$ ion activity in human breast cancer REI and mesothelioma H-MESO xenografts at normal and elevated blood glucose concentrations. O, Mean pH values of individual tumours at normoglycemia; ●, mean pH values of individual tumours at 5 h after the onset of glucose administration (PGC, 30 ± 3 mM).

*Effect of i.v. administration of mannitol on pH in human tumour xenografts*

In transplanted rodent tumours, parenteral administration of glucose is associated with a reduction of tumour blood flow (Kalmus *et al.*, 1989). A similar decrease of tumour perfusion can be induced by i.v. administration of the nonmetabolisable sugar mannitol. To investigate whether glucose-mediated reduction of tumour blood flow contributes to the acidic pH shift at elevated PGC, rats bearing endometrial carcinoma LA, lung cancer KO as well as breast cancers CH, BR and MX-1 were infused with a mannitol solution (0.2 g $ml^{-1}$) at the same dose rate as animals infused with glucose (~ 130 mg mannitol $kg^{-1}$ body weight $min^{-1}$ initially and 100 mg mannitol $kg^{-1}$ body weight $min^{-1}$ after 1 h). Tumour pH was measured at 5 h after initiation of mannitol infusion. The results are listed in Table II. In all xenograft lines investigated, mannitol caused only a slight shift of the pH histograms to move acidic values (on average by 0.04 pH units; statistically insignificant) whereas glucose administration resulted in pH reduction by 0.44 units ($P<0.0001$).

**Table II** Effect of i.v. mannitol or glucose infusion on pH in human tumour xenografts

| *Histopathological tumour type* | *Tumour pH*[a] *Untreated controls* | *Mannitol*[b] | *Glucose*[c] |
|---|---|---|---|
| Endometrial carcinoma LA | 6.79 ± 0.12 | 6.76 ± 0.09 | 6.23 ± 0.33 |
| Lung cancer KO | 6.97 ± 0.12 | 6.89 ± 0.10 | 6.64 ± 0.11 |
| Breast cancer BR | 6.69 ± 0.11 | 6.62 ± 0.08 | 6.40 ± 0.22 |
| Breast cancer CH | 6.88 ± 0.12 | 6.85 ± 0.13 | 6.48 ± 0.25 |
| Breast cancer MX-1 | 6.78 ± 0.14 | 6.77 ± 0.18 | 6.15 ± 0.27 |
| Mean | 6.82 | 6.78 | 6.38 |

[a]Mean values ± s.d. of 8–12 individual tumours per group. [b]Measured at 5 h after the onset of infusion of mannitol solution (0.2 g $ml^{-1}$) at a rate of 100 mg mannitol $kg^{-1}$ body weight $min^{-1}$. [c]Measured at 5 h after the initiation of glucose infusion (100 mg glucose $kg^{-1}$ body weight $min^{-1}$; PGC 30 ± 3 mM).

*Effect of i.v. glucose infusion on pH in normal tissues of tumour-bearing hosts*

Due to the size of the pH semi-microelectrodes used and other technical limitations, pH frequency distributions of human tumour xenografts could not be compared directly to pH histograms of the normal rat tissues corresponding to the respective types of tumours, e.g. mammary gland epithelium. Instead, pH was measured in three rat organs suited for insertion of pH semi-microelectrodes: liver, kidney and skeletal muscle. In contrast to the xenografted tumours, only a minor reduction of pH was observed in these normal rat tissues following high-dose i.v. glucose infusion (Table III). For example, the pH readings recorded in skeletal muscle covered a range of 6.89–7.54 (mean value, 7.22). This pH

**Table III** Effect of hyperglycemia on pH in normal tissues of rnu/rnu rats

| *Tissue* | *pH* *Normoglycemia (PGC, 6 ± 1 mM)* | *Hyperglycemia*[a] *(PGC, 30 ± 3 mM)* |
|---|---|---|
| Liver | 7.08 (6.79–7.48)[b] | 6.88 (6.64–7.12) |
| Kidney | 7.12 (6.74–7.38) | 6.96 (6.68–7.23) |
| Skeletal muscle | 7.22 (6.89–7.54) | 7.08 (6.73–7.36) |
| Mean | 7.14 | 6.97 |

[a]In hyperglycemic animals, measurements were performed at 4 h after PGC had reached a plateau. [b]Mean values with ranges in brackets.

frequency distribution showed only a slight shift to the left at 4 h after PGC had been raised to 30 ± 3 mM (mean pH value, 7.08; range 6.73–7.36; $P<0.05$). Similar results were obtained for kidney (Figure 7) and liver of tumour-bearing rnu/rnu rats (Table III).

*Systemic side effects of high-dose glucose infusion*

To confirm the safety of the glucose infusion regimen and to check for artifacts possibly caused by systemic disturbances induced by osmotic diuresis, we measured blood pressure, arterial blood gases, hemoglobin and protein concentrations as well as serum electrolytes in untreated and hyperglycemic rats. As summarised in Table IV temporary hyperglycemia had only marginal effects on hemodynamic parameters as well as indicators of fluid and electrolyte homeostasis. The decrease in arterial $pCO_2$ likely most is due to moderate hyperventilation of hyperglycemic rats (Volk *et al.*, 1993).

### Discussion

Carbohydrate metabolism of malignant cells is characterised by two distinct features: a high rate of glucose uptake and the formation of lactic acid. In mammalian cells the uptake of glucose is accomplished by a family of integral membrane proteins referred to as glucose transporters (Mueckler *et al.*, 1985; Gould & Bell, 1990). These proteins display cell type-specific patterns of expression, hormone-responsiveness and transport properties (Gould & Bell, 1990). In the process of malignant transformation, the number of glucose transporter molecules per cell is frequently upregulated (Birnbaum *et al.*, 1987; Flier *et al.*, 1987). This overall change is paralleled by alterations in the normally cell type-specific relative expression frequencies of various transporter subtypes, in favour of those transporters which are not insulin-dependent (Yamamoto *et al.*, 1990). As a result, glucose uptake into malignant cells is no longer regulated according to systemic or cellular demands, but almost exclusively dictated by the extracellular concentration of glucose (Eagle *et al.*, 1958). This property of cancer cells can be exploited to stimulate glucose uptake and, consecutively, lactic acid production selectively in malignant tissues by systemically increasing the concentration of glucose in the cellular microenvironment (Von Ardenne & Reitnauer, 1978; Jähde *et al.*, 1982*b*; Wike-Hooley *et al.*,1984; Ward & Jain, 1988; Hwang *et al.*, 1991; Jähde *et al.*, 1989).

**Figure 7** Effect of i.v. glucose infusion on pH in kidney of rnu/rnu rats: frequency distributions of single-point pH recordings. **a**, PGC, 6 ± 1 mM; 12 kidneys; number of pH readings, 147. **b**, PGC, 30 ± 3 mM; 13 kidneys; number of pH readings, 153.

**Table IV** Systemic effects of i.v. glucose infusion

| Parameter | *Normoglycemia (PGC, 6 ± 1 mM)* | *Hyperglycemia (PGC, 30 ± 3 mM)*[a] |
|---|---|---|
| MABP[b] (mmHg) | 98 ± 7[c] | 94 ± 6 |
| $pO_2$ (mmHg) | 93 ± 4.5 | 94 ± 2.0 |
| $pCO_2$ (mmHg) | 46 ± 0.5 | 31 ± 0.8 |
| pH | 7.36 ± 0.04 | 7.34 ± 0.05 |
| Hemoglobin ($g\,l^{-1}$) | 150 ± 9 | 159 ± 12 |
| $Na^+$ ($mmol\,l^{-1}$) | 141 ± 5 | 142 ± 4 |
| $K^+$ ($mmol\,l^{-1}$) | 5.6 ± 0.7 | 4.8 ± 0.9 |
| Protein ($g\,l^{-1}$) | 58 ± 3 | 58 ± 4 |

[a]Measured 5 h after onset of infusion of glucose. [b]Mean arterial blood pressure. [c]Mean values ± s.d. of 10 animals per group.

Without exception, the human tumour xenografts investigated in the present study responded to an increased PGC by an accumulation of acidic metabolites. In individual tumours, single-point pH readings as low as 5.47 were recorded. In seven xenograft lines, the mean pH was reduced below 6.25. On average, intratumoural pH was reduced to 6.43, a value corresponding to a ten-fold increase in the activity of $H^+$ ions as compared to arterial blood. This acidosis was tumour-specific. pH frequency distributions of liver, kidney and skeletal muscle of tumour-bearing hosts were only marginally affected by high-dose i.v. glucose infusion. As discussed previously (Jähde *et al.*, 1982*b*), these minor shifts of the pH histograms of normal tissues partly reflect organ-specific functional changes in tumour-bearing hosts during hyperglycemia, e.g. increased urine acidity in the case of kidney.

In view of the potential clinical use of pH-sensitive anticancer agents (Connors *et al.*, 1964; Yatvin *et al.*, 1980; Wike-Hooley *et al.*, 1984; Ward & Jain, 1988; Lavie *et al.*, 1991; Hiroaka & Hahn, 1989; Tannock & Rotin, 1989; Tietze *et al.*, 1989; Jähde *et al.*, 1989), we have analysed various parameters considered relevant to the design of treatment protocols. First, we asked whether or not four histopathological tumour entities of major clinical importance exhibited a similar pH response to glucose. On average, the pH values of breast, lung and gastrointestinal cancers, and sarcomas were reduced to 6.36, 6.53, 6.53 and 6.51, respectively, while the mean pH value of all other tumours analysed was reduced to 6.38. These results indicate that this manipulation of $H^+$ ion activity may be feasible in most human tumours requiring systemic treatment. Irrespective of the tissue of origin, there were, however, differences in the pH response to glucose between individual xenograft lines and also between individual tumours derived from a given xenograft line. For example, in the colon cancer line SW 707 hyperglycemia only reduced the mean pH from 6.98 to 6.78; but from mean values of 6.73 and 6.77, respectively, to 6.12 and 6.13 in pancreatic carcinoma STO and breast cancer SE. This hetereogeneity of the pH response, which in individual tumours could not be predicted from the $H^+$ ion activity at normoglycemia, is due to cell type-specific differences in the rate of lactic acid production as well as tissue-specific differences in the transport of glucose and acidic metabolites. In Warburg's classical studies on human tumour tissues *in vitro*, the metabolic quotient indicating lactic acid production in the presence of oxygen (aerobic glycolysis), $Q_{CO_2}^{O_2}$, varied by a factor of 5 between individual tumours (Warburg *et al.*, 1924), consistent with recent quantitations of lactate release from human tumour xenografts *in vivo* (Kallinowski *et al.*, 1989). Moreover, tumour blood flow, one of the most important determinants of glucose supply as well as lactic acid clearance, may vary by a factor of 10 among individual tumours of related histogenesis (Kallinowski *et al.*, 1989). As shown by Jähde *et al.* (1992), lactic acid production can be further increased by additional pharmacological interventions employing inorganic phosphate and m-iodobenzylguanidine in those tumours which exhibit only a moderate pH response to glucose. As demonstrated in N4 sarcomas and SCLC lung tumours, pH values of ~6.45 could be maintained for 24 h, a time interval exceeding the time required for activation of various pH-sensitive agents *in vitro* (Tietze *et al.*, 1989; Jähde *et al.*, 1989).

In MRI-H-221 xenografts, the mean pH was reduced to 6.46 when PGC was raised to 2.5-times the normal value. When blood glucose was further incresaed to 19 mM, tumour pH continued to decline; however, at PGC levels above 19 mM, the relative increment in acidity gradually levelled off, with minimum pH values recorded at a PGC of 30 mM. Even at this level of hyperglycemia no side effects (apart from osmotic diuresis) requiring interventions were observed as indicated by stable blood pressure, normal blood gases and serum electrolytes, a finding consistent with reports by other investigators (Krüger *et al.*, 1991). In healthy human volunteers as well as cancer patients, blood glucose levels ranging between 20 and 30 mM have been generated by high-dose i.v. glucose infusion (Förster, 1987; Krag *et al.*, 1990). Given adequate fluid volume replacement, this procedure was well tolerated. Since insulin secretion is not compromised in non-diabetic individuals, metabolic derangements characteristic of diabetic ketoacidosis do not occur following high-dose glucose infusion.

The activity of $H^+$ ions in tumour tissues depends on an interplay of various determinants. Among these, the rate of lactic acid production as well as the interstitial and vacular transport of glucose and lactate are of particular importance. The predictive value of the results presented here for the pH response to glucose of human tumours *in situ* depends, therefore, on the validity of xenografts as models of cellular metabolism as well as histomorphological parameters governing substrate and metabolite transport in tissues. Both *in vitro* and *in vivo*, human tumour cells, with rare exceptions, respond to elevated extracellular concentrations of glucose by increased lactic acid production (Naeslund & Swenson, 1953; Eagle *et al.*, 1958; Aisenberg, 1961; Ashby, 1966; Thistlethwaite, 1987; Lavie *et al.*, 1991). It is more difficult to predict whether acidic metabolites, once their production is stimulated, will indeed accumulate in human tumours *in situ* to a similar extent as observed in human tumour xenografts. The histomorphology of tumour xenografts does not always mirror the tissue architecture of primary tumours. In general, however, the vasculature of xenografted human tumours bears a high degree of resemblance in morphology as well as functional properties to the vascular network of primary tumours *in situ* (Vaupel *et al.*, 1987; Konerding *et al.*, 1989*a*; Konerding *et al.*, 1989*b*). It may thus be reasonably assumed that primary tumours will exhibit a similar pH response to glucose as their xenograft counterparts. This view is supported by pH measurements in human patients. As stated above, we are only aware of very few studies that have measured pH values in human primary tumours following stimulation of aerobic glycolysis. For example, in Ashby's series (1966), the average pH of 9 malignant melanomas was 6.57 ~ 40 min after PGC had been raised to 23 mM.

pH measurements performed with semi-microelectrodes primarily reflect $H^+$ ion activities in the extracellular space although, by destruction of cells, various intracellular compartments may contribute to pH readings ('aggregate' pH) (Jähde *et al.*, 1982*b*). The cytotoxic effects of pH-sensitive agents, on the other hand, are either dependent on extracellular or on intracellular $H^+$ ion activity. For example, acid-labile prodrugs and pH-sensitive immunoconjugates may be activated in the interstitial space whereas the cytotoxicity of alkylating drugs and hyperthermia is sensitive to changes in intracellular pH. Although mammalian cells are able to maintain pH gradients across plasma membranes, marked alterations of extracellular pH are accompanied by parallel, although not fully equivalent shifts of intracellular pH (Tannock & Rotin, 1989; Jähde *et al.*, 1989). The pH measurements reported here, therefore, are likely to indicate not only an acidification of tumour interstitial fluid following stimulation of aerobic glycolysis, but also a shift of intracellular pH. This is supported by measurements of intracellular pH following stimulation of aerobic glycolysis with the use of nuclear magnetic resonance spectroscopy *in vitro* and *in vivo* (Desmoulin *et al.*, 1986). For example, Hwang *et al.* (1991) demonstrated a reduction of predominantly intracellular pH by 0.79 units in RIF-1 tumours following i.p. glucose administration. Thus, even though at normoglycemia malignant cells may be capable to maintain the intracellular pH within normal limits vis-à-vis a slightly acidic microenvironment (Griffiths, 1991), present evidence suggests that, by metabolic manipulations, not only the extracellular, but also intracellular pH can be reduced.

Tumour-selective modification of the cellular microenvironment deserves particular attention as a general approach to the development of more effective treatment modalities for solid tumours. As indicated by the present study, a selective increase in $H^+$ ion activity in malignant tissues can be induced in a wide variety of malignant tumours of diverse histogenesis. Glucose, the biochemical response modifier used, in nontoxic (Förster, 1987; Krag *et al.*, 1990). Moreover, $H^+$ ions are potent modulators of various types of chemical reactions. Several classes of anti-cancer agents with different mechanisms of action, as diverse as alkylating drugs, pH-sensitive immunoconjugates or hyperthermia, have been identified whose cytotoxicity is sensitive to changes in microenvironmental pH (Yatvin *et al.*, 1980; Lavie *et al.*, 1991; Hiroaka & Hahn, 1989; Jähde *et al.*, 1989). Acid-labile prodrugs of cytotoxic agents have been synthesised which are stable, and hence nontoxic at physiological pH (Tietze *et al.*, 1989; Glüsenkamp *et al.*, 1992). At slightly acidic pH, these prodrugs decompose with the liberation of potent cytotoxic species. Thus, at pH 6.4 (i.e. the mean pH measured in human tumour xenografts following glucose-stimulated the present aerobic glycolysis) the potency of 'second generation' acid-labile prodrugs, as expressed in terms of drug concentrations required to achieve equivalent cell killing *in vitro*, increases by a factor of 10–20 as compared to pH 7.4 (Glüsenkamp *et al.*, 1992). We believe, therefore, that the results presented here encourage further exploration of this approach.

This work was supported by Dr Mildred Scheel Stiftung für Krebsforschung (W 15/88 Ra 3). We are indebted to Drs V. Budach (Essen), H.H. Fiebig (Freiburg i.Br), A. Schmidt-Matthiesen (Frankfurt a.M.), K. Wayss (Heidelberg), and H.J.C. Wenisch (Frankfurt a.M.) for donating human tumour xenografts and to Dr R. Scherer (Essen) for providing blood pressure monitoring equipment. We also thank M. Zaczek and T. Kämper for expert technical assistance and Dipl.-Ing. K. Lennartz for preparation of the charts.

### References

AISENBERG, A.C. (1961). *The Glycolysis and Respiration of Tumors.* Academic Press: New York and London.

ASHBY, B.S. (1966). pH-Studies in human malignant tumours. *Lancet*, **2**, 312–315.

BIRNBAUM, M.J., HASPEL, H.C. & ROSEN, O.M. (1987). Transformation of rat fibroblasts by FSV rapidly increases glucose transporter gene transcription. *Science (Washington DC)*, **235**, 1495–1498.

BUSCH, H. (1990). The final common pathway of cancer: presidential address. *Cancer Res.*, **50**, 4830–4838.

CONNORS, T.A., MITCHLEY, B.C.V., ROSENOER, V.M. & ROSS, W.C.J. (1964). The effect of glucose pretreatment on the carcinostatic and toxic activities of some alkylating agents. *Biochem. Pharmacol.*, **13**, 395–400.

DESMOULIN, F., GALONS, J.-P., CANIONI, P., MARVALDI, J. & COZZONE, P.J. (1986). $^{31}P$ Nuclear magnetic resonance study of a human colon adenocarcinoma cultured cell line. *Cancer Res.*, **46**, 3768–3774.

EAGLE, H., BARBAN, S., LEVY, M. & SCHULZE, H.O. (1958). The utilization of carbohydrates by human cell cultures. *J. Biol. Chem.*, **233**, 551–558.

FLIER, J.S., MUECKLER, M.M., USHER, P. & LODISH, H.F. (1987). Elevated levels of glucose transport and transporter messenger RNA are induced by ras and sarc oncogenes. *Science (Washington DC)*, **235**, 1492–1495.

FÖRSTER, H. (1987). Fruktose und Sorbit als energieliefernde Substrate für die parenterale Ernährung. *Infusionstherapie*, **14**, 98–109.

GLÜSENKAMP, K.-H., JÄHDE, E., MENGEDE, C., DROSDZIOK, W. & RAJEWSKY, M.F. (1992). Computer-aided design of non-toxic prodrugs for tumor-selective activation by acid catalysis. *Ann. Oncol.*, **3** (Suppl. 1), 87.

GOULD, G.W. & BELL, G.I. (1990). Facilitative glucose transporters: an expanding family. *Trends Biol. Sci.*, **15**, 18–23.

GRIFFITHS, J.R. (1991). Are cancer cells acidic? *Br. J. Cancer*, **64**, 425–427.

HIROAKA, M. & HAHN, G.M. (1989). Comparison between tumor pH and cell sensitivity to heat in RIF-1 tumors. *Cancer Res.*, **49**, 3734–3736.

HWANG, Y.C., KIM, S.-G., EVELHOCH, J.L., SEYEDSADR, M. & ACKERMANN, J.H. (1991). Modulation of murine radiation-induced fibrosarcoma-1 tumor metabolism and blood flow *in situ* via glucose and mannitol administration monitored by $^{31}P$ and $^{2}H$ nuclear magnetic resonance spectroscopy. *Cancer Res.*, **51**, 3108–3118.

JÄHDE, E. & RAJEWSKY, M.F. (1982*a*). Tumor-selective modification of cellular microenvironment *in vivo*: effect of glucose infusion on the pH in normal and malignant rat tissues. *Cancer Res.*, **42**, 1505–1512.

JÄHDE, E., BAUMGÄRTL, H. & RAJEWSKY, M.F. (1982*b*). pH Distributions in transplanted neural tumors and normal tissues of BD IX rats as measured with microelectrodes. *Cancer Res.*, **42**, 1498–1504.

JÄHDE, E., GLÜSENKAMP, K.-H., KLÜNDER, I., HÜLSER, D.F., TIETZE, L.-F. & RAJEWSKY, M.F. (1989). Hydrogen ion-mediated enhancement of cytotoxicity of bis-chloroethylating drugs in rat mammary carcinoma cells *in vitro*. *Cancer Res.*, **49**, 2965–2972.

JÄHDE, E., VOLK, T., ATEMA, A., SMETS, L.A., GLÜSENKAMP, K.-H., RAJEWSKY, M.F. (1992). pH in human tumor xenografts and transplanted rat tumors: effect of insulin, inorganic phosphate, and m-iodobenzylguanidine. *Cancer Res.*, **52**, 6209–6215.

KALLINOWSKI, F., SCHLENGER, K.H., RUNKEL, S., KLOES, M., STOHRER, M., OKUNIEFF, P. & VAUPEL, P. (1989). Blood flow, metabolism, cellular microenvironment, and growth rate of human tumor xenografts. *Cancer Res.*, **49**, 3759–3764.

KALMUS, J., OKUNIEFF, P. & VAUPEL, P. (1989). Effect of intraperitoneal versus intravenous glucose administration on laser doppler flow in murine FSaII tumors and normal skin. *Cancer Res.*, **49**, 6313–6317.

KONERDING, M.A., STEINBERG, F. & STREFFER, C. (1989*a*). The vasculature of xenotransplanted human melanomas and sarcomas. 1. Vascular corrosion casting studies. *Acta Anat.*, **136**, 21–26.

KONERDING, M.A., STEINBERG, F. & STREFFER, C. (1989*b*). The vasculature of xenotransplanted human melanomas and sarcomas. 2. Scanning and transmission electron microscopic studies. *Acta Anat.*, **136**, 27–32.

KRAG, D.N., STORM, F.K. & MORTON, D.L. (1990). Induction of transient hyperglycemia in cancer patients. *Int. J. Hyperthermia*, **6**, 741–744.

KRÜGER, W., MAYER, W.-K., SCHAEFER, C., STOHRER, M. & VAUPEL, P. (1991). Acute changes of systemic parameters in tumour-bearing rats, and of tumour glucose, lactate, and ATP levels upon local hyperthermia and/or hyperglycaemia. *J. Cancer Res. Clin. Oncol.*, **117**, 409–415.

LAVIE, E., HIRSCHBERG, D.L., SCHREIBER, G., THOR, K., HILL, L., HELLSTROM, I. & HELLSTROM, K.-E. (1991). Monoclonal antibody L6-daunomycin conjugates constructed to release free drug at the lower pH of tumor tissue. *Cancer Immunol. Immunother.*, **33**, 223–230.

LIPPMANN, H.G. & GRAICHEN, D. (1977). Glukose- und $K^+$-Bilanz während hochdosierter intravenöser Glukosezufuhr. *Infusionstherapie*, **4**, 166–178.

LYON, R.C., COHEN, J.S., FAUSTINO, P.J., MEGNIN, F. & MYERS, C.E. (1988). Glucose metabolism in drug-sensitive and drug-resistant human breast cancer cells monitored by magnetic resonance spectroscopy. *Cancer Res.*, **48**, 870–877.

MUECKLER, M., CARUSO, C., BALDWIN, S.A., PANICO, M., BLENCH, I., MORRIS, H.R., ALLARD, W.J., LEINHARD, G.E. & LODISH, H.F. (1985). Sequence and structure of a human glucose transporter. *Science (Washington DC)*, **229**, 941–945.

NAESLUND, J. & SWENSON, K.-E. (1953). Investigations on the pH of malignant tumours in mice and humans after the administration of glucose. *Acta Obstet. Gynecol. Scan.*, **32**, 359–367.

SCHUTZBANK, T., ROBINSON, R., OREN, M. & LEVINE, A.J. (1982). SV40 large tumor antigen can regulate some cellular transcripts in a positive fashion. *Cell*, **30**, 481–490.

TANNOCK, I.F. & ROTIN, D. (1989). Acid pH in tumors and its potential for therapeutic exploitation. *Cancer Res.*, **49**, 4373–4384.

THISTLETHWAITE, A.J., ALEXANDER, G.A., MOYLAN III, D.J. & LEEPER, D.B. (1987). Modification of human tumor pH by elevation of blood glucose. *Int. J. Radiat. Oncol. Biol. Phys.*, **13**, 603–610.

TIETZE, L.F., NEUMANN, M., MÖLLERS, T., FISCHER, R., GLÜSENKAMP, K.-H., RAJEWSKY, M.F. & JÄHDE, E. (1989). Proton-mediated liberation of aldophosphamide from a nontoxic prodrug: a strategy for tumor-selective activation of cytocidal drugs. *Cancer Res.*, **49**, 4179–4184.

VAUPEL, P., FORTMEYER, H.P., RUNKEL, S. & KALLINOWSKI, F. (1987). Blood flow, oxygen consumption, and tissue oxygenation of human breast cancer xenografts in nude rats. *Cancer Res.*, **47**, 3496–3503.

VAUPEL, P., KALLINOWSKI, F. & OKUNIEFF, P. (1989). Blood flow, oxygen and nutrient supply, and metabolic microenvironment of human tumors: a review. *Cancer Res.*, **49**, 6449–6465.

VOLK, T., ROSZINSKI, S., JÄHDE, E., GLÜSENKAMP, K.-H. & RAJEWSKY, M.F. (1993). Effect of glucose-mediated pH reduction and cyclophosphamide on oxygenation of transplanted rat tumors. *Int. J. Radiat. Oncol. Biol. Phys.*, **25**, 465–471.

VON ARDENNE, M. & REITNAUER, P.G. (1978). Über manipulierte Übersäuerung autochthoner Tumoren. *Onkologie*, **1**, 85–88.

WARBURG, O., POSENER, K. & NEGELEIN, E. (1924). Über den Stoffwechsel der Carcinomzelle. *Biochem. Z.*, **152**, 309–344.

WARD, K.A. & JAIN, R.K. (1988). Response of tumours to hyperglycemia: characterization, significance and role in hyperthermia. *Int. J. Hyperthermia*, **4**, 223–250.

WEBER, G. (1977). Enzymology of cancer cells. *New Engl. J. Med.*, **296**, 541–551.

WIKE-HOOLEY, J.L., HAVEMANN, J. & REINHOLD, H.S. (1984). The relevance of tumour pH to the treatment of malignant disease. *Radiother. Oncol.*, **2**, 343–366.

YAMAMOTO, T., SEINO, Y., FUKUMOTO, H., KOH, G., YANO, H., INAGAKI, N., YAMADA, Y., INOUE, K., MANABE, T. & IMURA, H. (1990). Over-expression of facilitative glucose transporter genes in human cancer. *Biochem. Biophys. Res. Commun.*, **170**, 223–230.

YATVIN, M.B., KREUTZ, W., HORWITZ, B.A. & SHINITZKY, M. (1980). pH-Sensitive liposomes: possible clinical implications. *Science (Washington DC)*, **210**, 1253–1254.


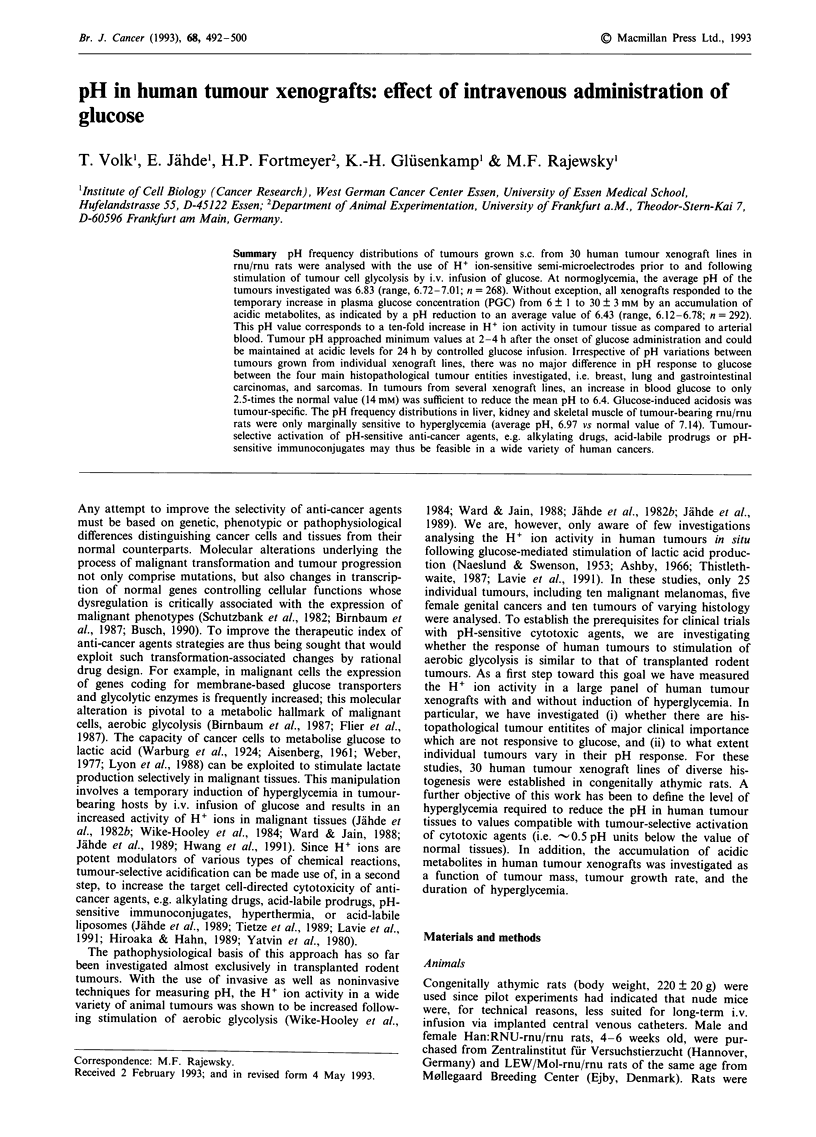

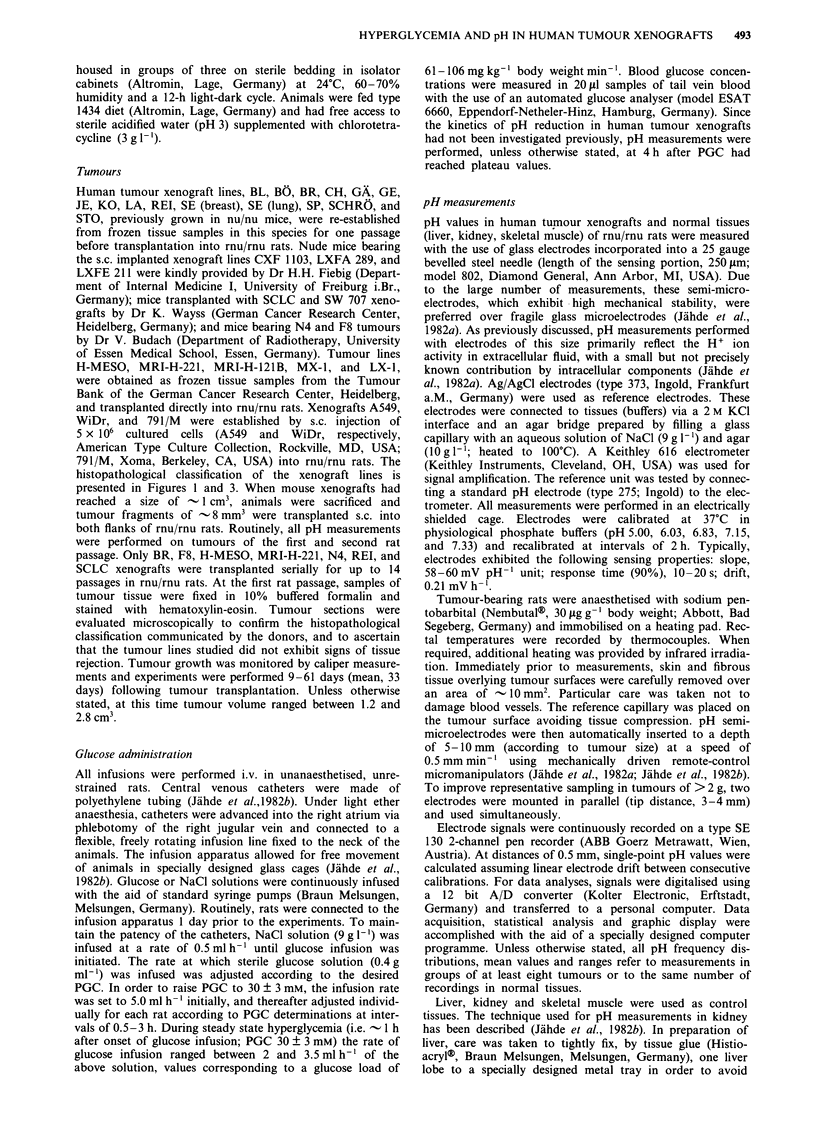

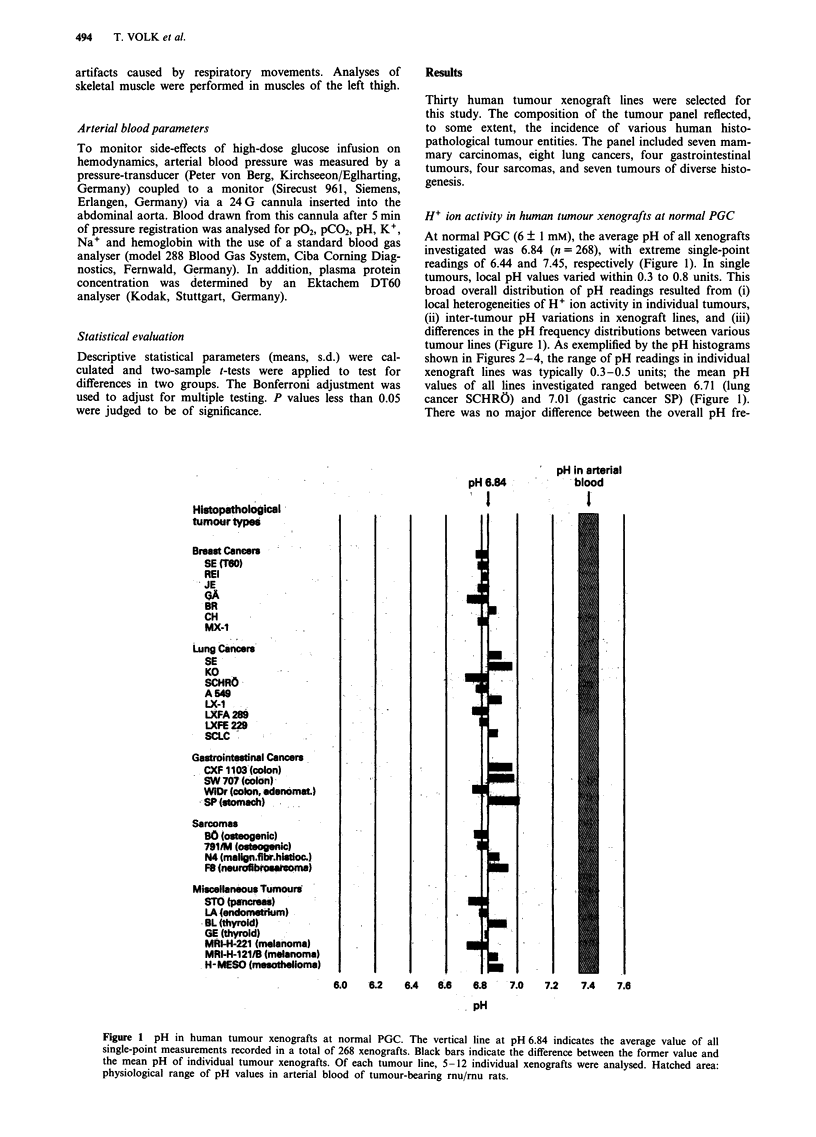

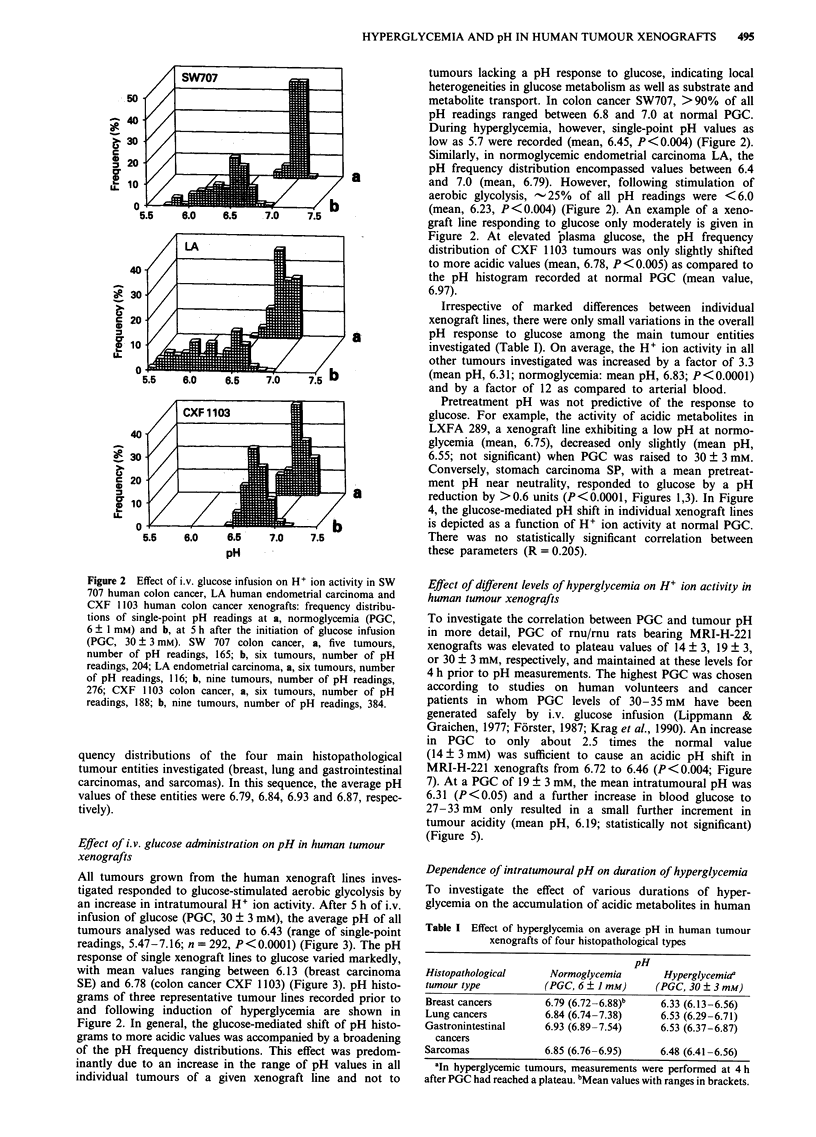

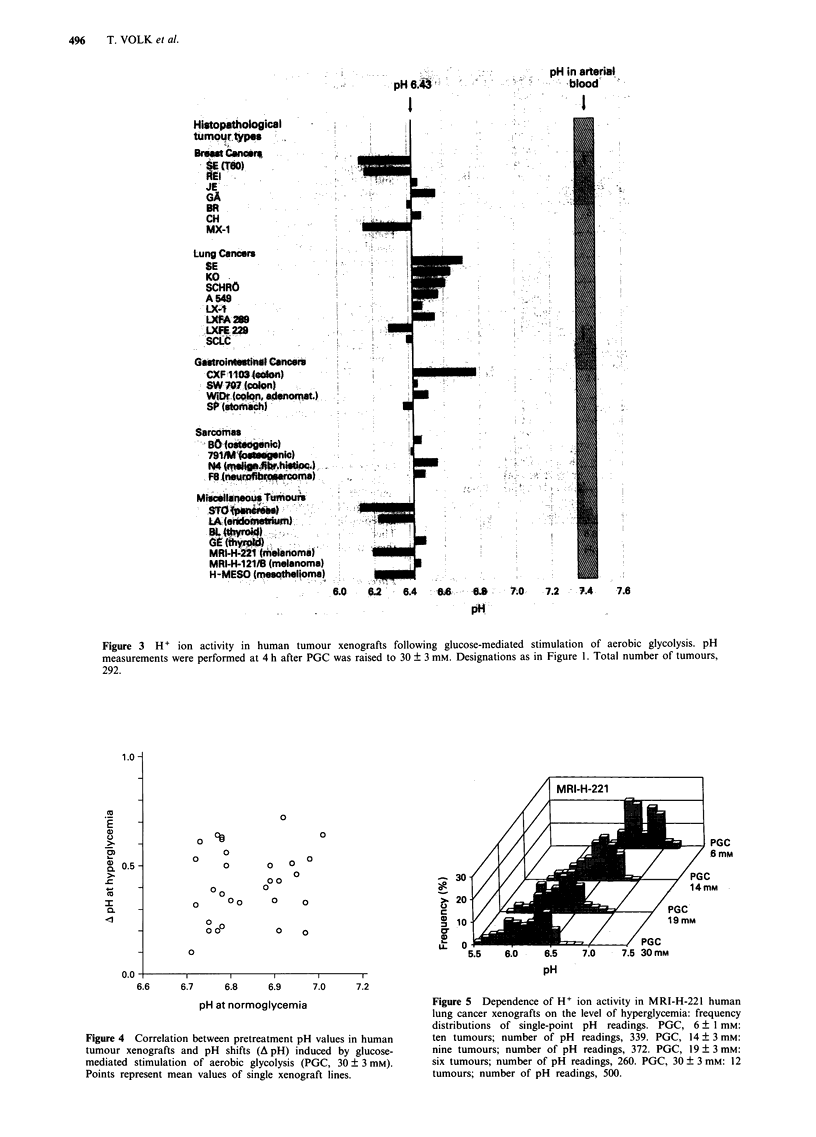

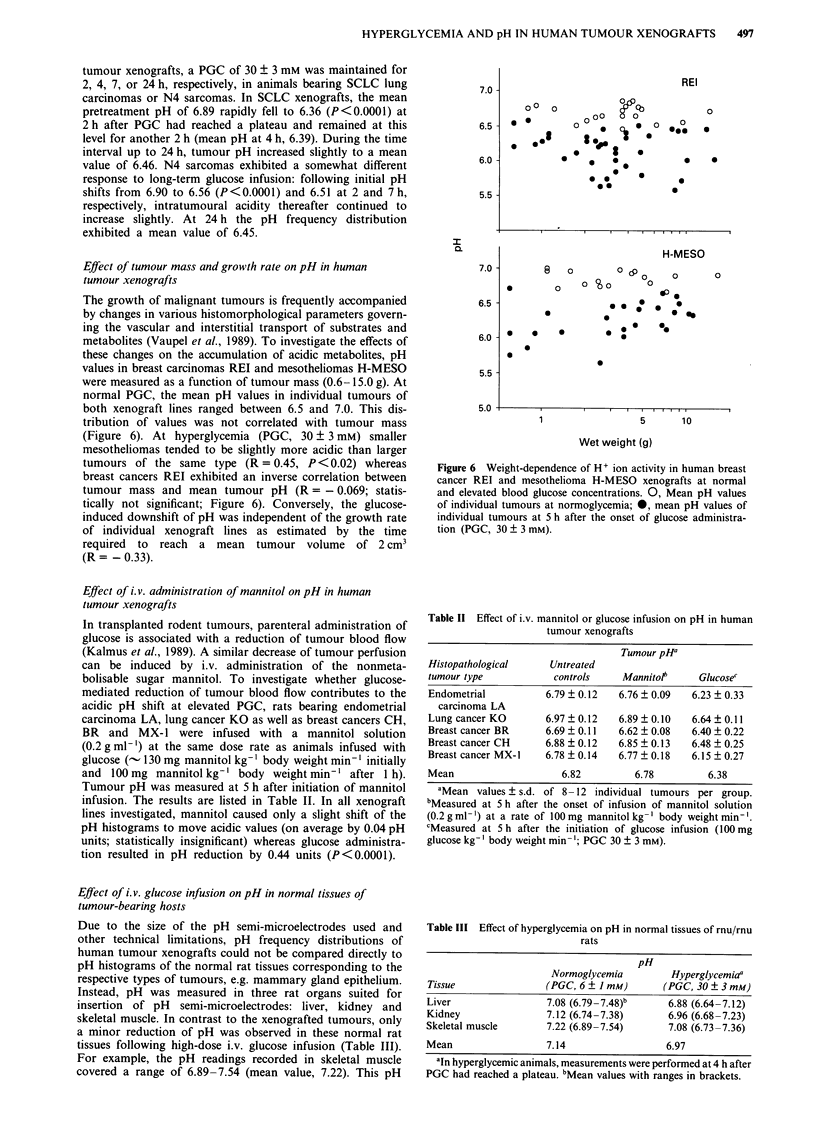

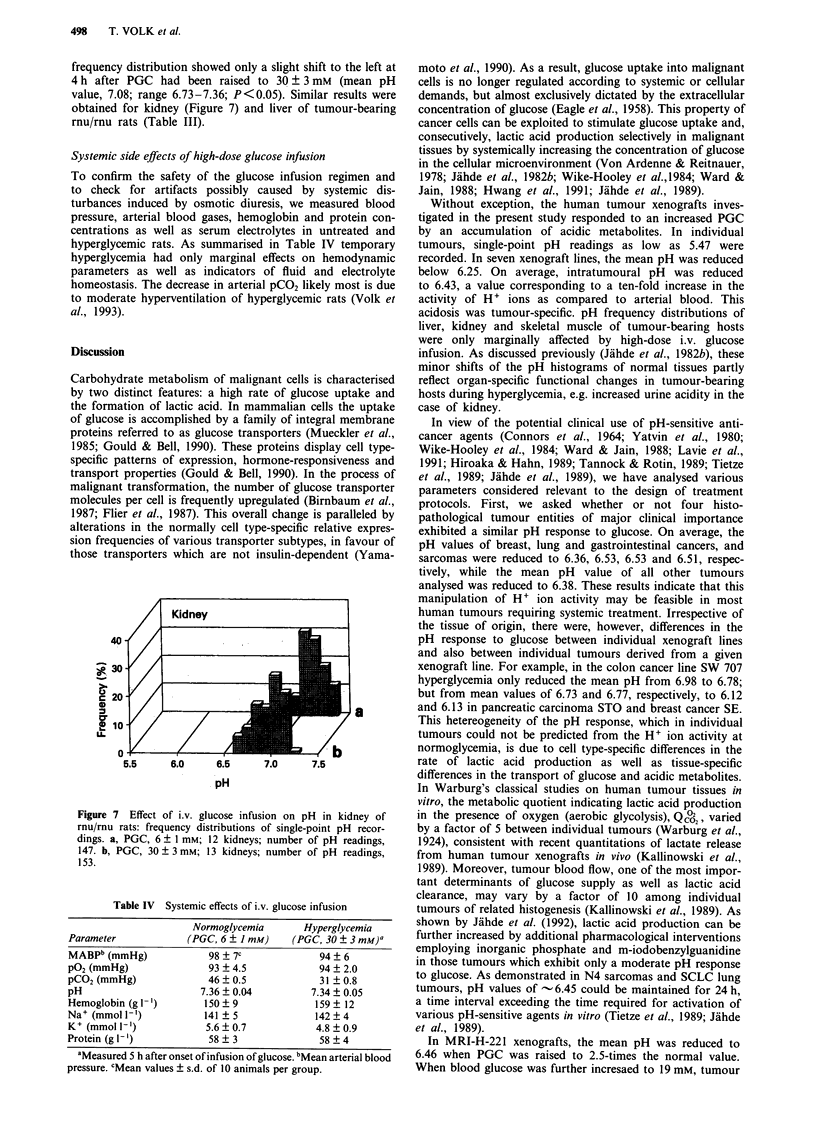

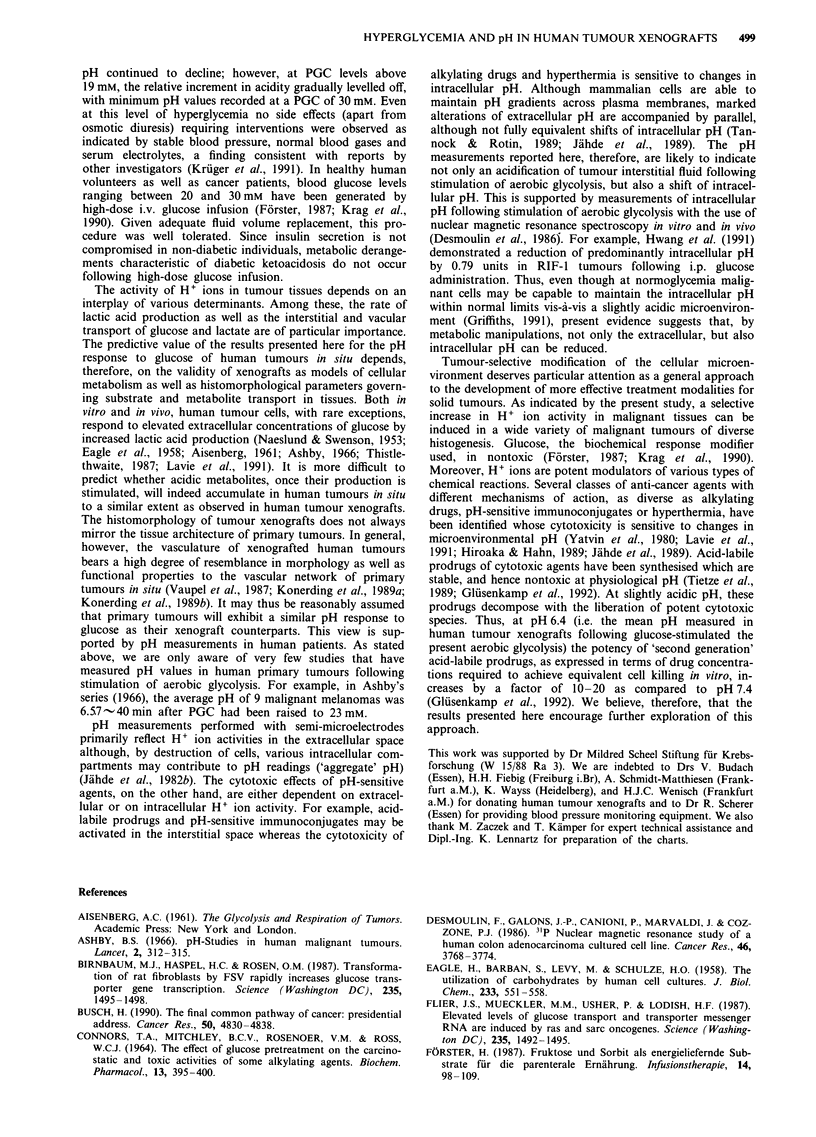

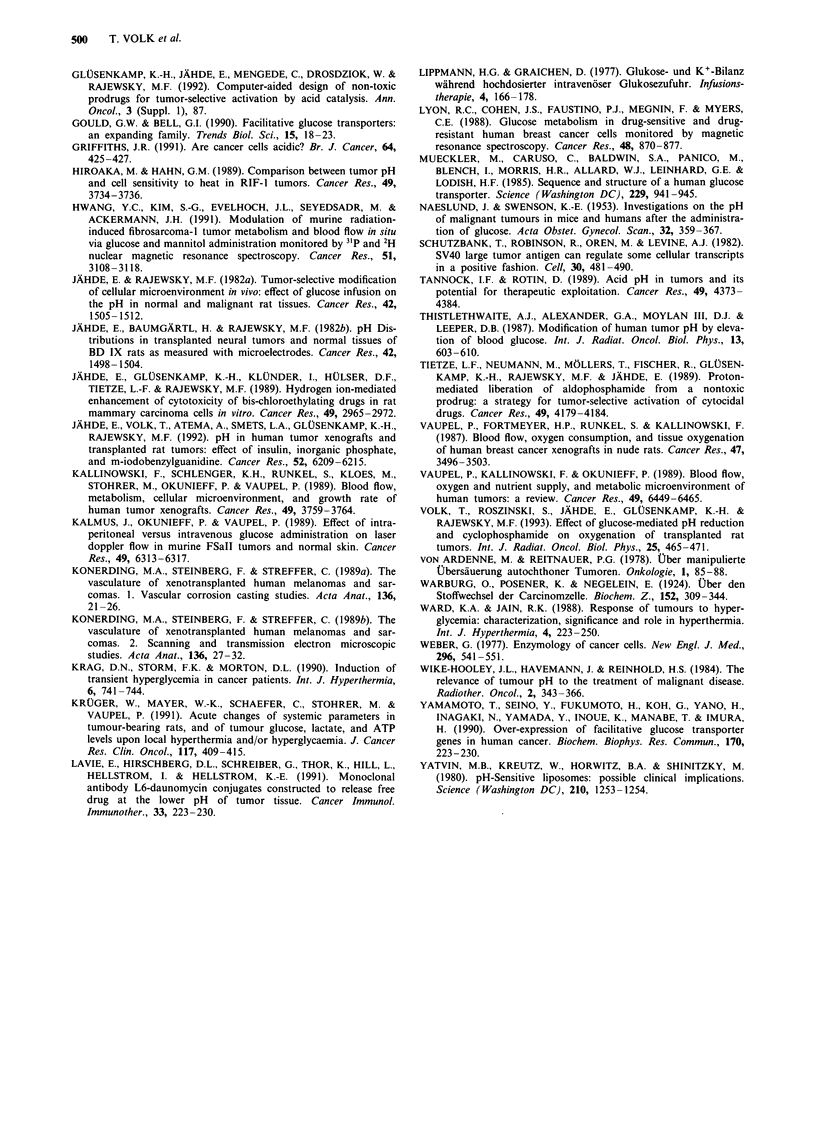


## References

[OCR_01345] Ashby B. S. (1966). pH studies in human malignant tumours.. Lancet.

[OCR_01349] Birnbaum M. J., Haspel H. C., Rosen O. M. (1987). Transformation of rat fibroblasts by FSV rapidly increases glucose transporter gene transcription.. Science.

[OCR_01355] Busch H. (1990). The final common pathway of cancer.. Cancer Res.

[OCR_01359] CONNORS T. A., MITCHLEY B. C., ROSENOER V. M., ROSS W. C. (1964). THE EFFECT OF GLUCOSE PRETREATMENT ON THE CARCINOSTATIC AND TOXIC ACTIVITIES OF SOME ALKYLATING AGENTS.. Biochem Pharmacol.

[OCR_01367] Desmoulin F., Galons J. P., Canioni P., Marvaldi J., Cozzone P. J. (1986). 31P nuclear magnetic resonance study of a human colon adenocarcinoma cultured cell line.. Cancer Res.

[OCR_01371] EAGLE H., BARBAN S., LEVY M., SCHULZE H. O. (1958). The utilization of carbohydrates by human cell cultures.. J Biol Chem.

[OCR_01376] Flier J. S., Mueckler M. M., Usher P., Lodish H. F. (1987). Elevated levels of glucose transport and transporter messenger RNA are induced by ras or src oncogenes.. Science.

[OCR_01382] Förster H. (1987). Fruktose und Sorbit als energieliefernde Substrate für die parenterale Ernährung.. Infusionsther Klin Ernahr.

[OCR_01395] Gould G. W., Bell G. I. (1990). Facilitative glucose transporters: an expanding family.. Trends Biochem Sci.

[OCR_01399] Griffiths J. R. (1991). Are cancer cells acidic?. Br J Cancer.

[OCR_01403] Hiraoka M., Hahn G. M. (1989). Comparison between tumor pH and cell sensitivity to heat in RIF-1 tumors.. Cancer Res.

[OCR_01408] Hwang Y. C., Kim S. G., Evelhoch J. L., Seyedsadr M., Ackerman J. J. (1991). Modulation of murine radiation-induced fibrosarcoma-1 tumor metabolism and blood flow in situ via glucose and mannitol administration monitored by 31P and 2H nuclear magnetic resonance spectroscopy.. Cancer Res.

[OCR_01428] Jähde E., Glüsenkamp K. H., Klünder I., Hülser D. F., Tietze L. F., Rajewsky M. F. (1989). Hydrogen ion-mediated enhancement of cytotoxicity of bis-chloroethylating drugs in rat mammary carcinoma cells in vitro.. Cancer Res.

[OCR_01422] Jähde E., Rajewsky M. F., Baumgärtl H. (1982). pH distributions in transplanted neural tumors and normal tissues of BDIX rats as measured with pH microelectrodes.. Cancer Res.

[OCR_01416] Jähde E., Rajewsky M. F. (1982). Tumor-selective modification of cellular microenvironment in vivo: effect of glucose infusion on the pH in normal and malignant rat tissues.. Cancer Res.

[OCR_01433] Jähde E., Volk T., Atema A., Smets L. A., Glüsenkamp K. H., Rajewsky M. F. (1992). pH in human tumor xenografts and transplanted rat tumors: effect of insulin, inorganic phosphate, and m-iodobenzylguanidine.. Cancer Res.

[OCR_01439] Kallinowski F., Schlenger K. H., Runkel S., Kloes M., Stohrer M., Okunieff P., Vaupel P. (1989). Blood flow, metabolism, cellular microenvironment, and growth rate of human tumor xenografts.. Cancer Res.

[OCR_01445] Kalmus J., Okunieff P., Vaupel P. (1989). Effect of intraperitoneal versus intravenous glucose administration on laser Doppler flow in murine FSaII tumors and normal skin.. Cancer Res.

[OCR_01451] Konerding M. A., Steinberg F., Streffer C. (1989). The vasculature of xenotransplanted human melanomas and sarcomas on nude mice. I. Vascular corrosion casting studies.. Acta Anat (Basel).

[OCR_01457] Konerding M. A., Steinberg F., Streffer C. (1989). The vasculature of xenotransplanted human melanomas and sarcomas on nude mice. II. Scanning and transmission electron microscopic studies.. Acta Anat (Basel).

[OCR_01463] Krag D. N., Storm F. K., Morton D. L. (1990). Induction of transient hyperglycaemia in cancer patients.. Int J Hyperthermia.

[OCR_01468] Krüger W., Mayer W. K., Schaefer C., Stohrer M., Vaupel P. (1991). Acute changes of systemic parameters in tumour-bearing rats, and of tumour glucose, lactate, and ATP levels upon local hyperthermia and/or hyperglycaemia.. J Cancer Res Clin Oncol.

[OCR_01475] Lavie E., Hirschberg D. L., Schreiber G., Thor K., Hill L., Hellstrom I., Hellstrom K. E. (1991). Monoclonal antibody L6-daunomycin conjugates constructed to release free drug at the lower pH of tumor tissue.. Cancer Immunol Immunother.

[OCR_01482] Luppmann H. G., Graichen D. (1977). Glukose- und K+-Bilanz während hochdosierter intravenöser Glukosezufuhr.. Infusionsther Klin Ernahr.

[OCR_01487] Lyon R. C., Cohen J. S., Faustino P. J., Megnin F., Myers C. E. (1988). Glucose metabolism in drug-sensitive and drug-resistant human breast cancer cells monitored by magnetic resonance spectroscopy.. Cancer Res.

[OCR_01493] Mueckler M., Caruso C., Baldwin S. A., Panico M., Blench I., Morris H. R., Allard W. J., Lienhard G. E., Lodish H. F. (1985). Sequence and structure of a human glucose transporter.. Science.

[OCR_01499] NAESLUND J., SWENSON K. E. (1953). Investigations on the pH of malignant tumors in mice and humans after the administration of glucose.. Acta Obstet Gynecol Scand.

[OCR_01504] Schutzbank T., Robinson R., Oren M., Levine A. J. (1982). SV40 large tumor antigen can regulate some cellular transcripts in a positive fashion.. Cell.

[OCR_01509] Tannock I. F., Rotin D. (1989). Acid pH in tumors and its potential for therapeutic exploitation.. Cancer Res.

[OCR_01516] Thistlethwaite A. J., Alexander G. A., Moylan D. J., Leeper D. B. (1987). Modification of human tumor pH by elevation of blood glucose.. Int J Radiat Oncol Biol Phys.

[OCR_01522] Tietze L. F., Neumann M., Möllers T., Fischer R., Glüsenkamp K. H., Rajewsky M. F., Jähde E. (1989). Proton-mediated liberation of aldophosphamide from a nontoxic prodrug: a strategy for tumor-selective activation of cytocidal drugs.. Cancer Res.

[OCR_01527] Vaupel P., Fortmeyer H. P., Runkel S., Kallinowski F. (1987). Blood flow, oxygen consumption, and tissue oxygenation of human breast cancer xenografts in nude rats.. Cancer Res.

[OCR_01533] Vaupel P., Kallinowski F., Okunieff P. (1989). Blood flow, oxygen and nutrient supply, and metabolic microenvironment of human tumors: a review.. Cancer Res.

[OCR_01538] Volk T., Roszinski S., Jähde E., Glüsenkamp K. H., Rajewsky M. F. (1993). Effect of glucose-mediated pH reduction and cyclophosphamide on oxygenation of transplanted rat tumors.. Int J Radiat Oncol Biol Phys.

[OCR_01552] Ward K. A., Jain R. K. (1988). Response of tumours to hyperglycaemia: characterization, significance and role in hyperthermia.. Int J Hyperthermia.

[OCR_01557] Weber G. (1977). Enzymology of cancer cells (second of two parts).. N Engl J Med.

[OCR_01561] Wike-Hooley J. L., Haveman J., Reinhold H. S. (1984). The relevance of tumour pH to the treatment of malignant disease.. Radiother Oncol.

[OCR_01566] Yamamoto T., Seino Y., Fukumoto H., Koh G., Yano H., Inagaki N., Yamada Y., Inoue K., Manabe T., Imura H. (1990). Over-expression of facilitative glucose transporter genes in human cancer.. Biochem Biophys Res Commun.

[OCR_01573] Yatvin M. B., Kreutz W., Horwitz B. A., Shinitzky M. (1980). pH-sensitive liposomes: possible clinical implications.. Science.

[OCR_01544] von Ardenne M., Reitnauer P. G. (1978). Uber manipulierte Ubersäuerung autochthoner Tumoren.. Onkologie.

